# Sustainable Functional Food Processing

**DOI:** 10.3390/foods10071438

**Published:** 2021-06-22

**Authors:** Predrag Putnik, Danijela Bursać Kovačević

**Affiliations:** 1 Department of Food Technology, University North, Trg dr. Žarka Dolinara 1, 48000 Koprivnica, Croatia; 2 Department of Food Engineering, Faculty of Food Technology and Biotechnology, University of Zagreb, Pierottijeva 6, 10000 Zagreb, Croatia

Functional nutrition has become one of the main directions for a healthy lifestyle and sustainable food production due to its promising positive influence on health and its association with the use of raw materials of natural origin [1,2,3]. Therefore, it has attracted great interest from both consumers and manufacturers concerned about human well-being and sustainable economic growth [4]. Not surprisingly, new socio-demographic trends (e.g., longer life expectancy, promotion of healthy lifestyles, better healthcare, etc.) supported functional sector to become an increasingly lucrative segment of the food industry with a rapidly growing market [5].

Functional foods are industrially processed or unprocessed natural foods that have beneficial health effects beyond their basic nutritional value when consumed regularly [6]. Consumers today are increasingly looking for products that are safe, natural, have Generally Recognized as Safe (GRAS) status, and are produced using sustainable and/or ecologically sound technologies. For this reason, “functional food” is an increasingly popular term in the social and scientific spheres, so the industry is continuously investing in the development of a sector that can offer products with additional benefits for consumer health. Here, it is important to emphasize that clinical trials (randomized, double-blind, and placebo-controlled) should be conducted for the foods that are to be labeled as “functional” in order to draw conclusions about the health benefits of the products [6].

For instance, functional juices and other beverages produced from indigenous fruits (which are economically poorly explored) represent an interesting niche, with all the above characteristics to satisfy the interests of different food markets [7,8]. Accordingly, recent trends in the juice and beverage industry are aimed at producing functional juices and beverages with various raw materials such as vitamins and their precursors; minerals; fiber; unsaturated fatty acids; biologically active compounds (BACs), including polyphenols, carotenoids, chlorophylls, tannins, etc.; various antioxidants; probiotics; and prebiotics [9,10]. Due to their considerable nutritional value, fruit juices have been found to be excellent carriers or transport vehicles for probiotic bacteria.

Here, the focus is on functional ingredients such as BACs [11] and probiotics [12], which are responsible for numerous beneficial effects of functional foods on health. Unfortunately, the majority of BACs are thermolabile, which is particularly important for food production, where classical heat treatments (e.g., pasteurization) are still used. As thoroughly documented, this leads to food degradation and affects the quality of the final product. In order to prevent such negative effects of the production process, scientists and engineers have focused on developing economical and environmentally friendly technologies capable of maintaining the nutritional and sensory quality of the food as well as microbiological stability during functional food processing [13]. Such approaches are based on low energy consumption and on the use of low-impact processing and “hurdle technology,” combining advanced (e.g., high power ultrasound (HPU), pulsed electric field (PEF), high pressure processing (HPP), etc.) and conventional food technologies, e.g., pasteurization [12,14,15]. Moreover, aside from food quality and safety, food design is also important for sensory appeal for consumers and economic success. Hence, technologies such as 3D food printing can be particularly useful in functional food production [16]. In the food industry, 3D printing is being explored in many areas, such as personalized and digitized nutrition, supply chain simplification, and expanding food offerings.

Aside from above mentioned, production of functional foods with application of innovative technologies is becoming increasingly popular (e.g., juices, dairy etc.) due to growing governmental requirements and support to decrease high expenditure and disposal of toxic chemicals and energy [12]. Since raw materials used for production of functional foods commonly employ suitable raw materials (fruits, vegetables, legumes etc.) they are mixed well with probiotics and/or biologically active compounds from plant and/or animal origin [10,16,17,18,19,20,21,22]. General direction for production of such foods is to have adherence to local diets (e.g., Mediterranean diets) while supplying local food markets with healthy alternatives for consumers. To that end it is encouraging existence, at (inter-)national levels (e.g., Research Executive Agency from European Commission; Croatian Science Foundation etc.), of initiatives to financially support projects able to develop nutritious foods with sustainable processing while overcoming limitations associated with upscaling of advanced technology in manufacturing. For instance, good example represents European SFS-funding program or 3D-SustJuice project funded by the Croatian Science Foundation (Hurdle technology and 3D printing for sustainable fruit juice processing and preservation) which is strongly related to this special issue and serves as a vehicle for dissemination of important data to scientific community [23]. Consequently, this Special Issue of *Foods* collected data relevant to sustainable functional food production [1,10,12,17,22,24,25,26], hurdle technology [12], advanced food processing [1,2,12,17,24,25,26,27,28,29,30,31,32], functional beverages [1,12,17,18,25,33], probiotics and BACs [1,2,12,17,20,21,22,24,25,26,27,28,29,30,31,32], and authentic fruits [7,25,30].

For this valuable collection of data, editors would like to thank all the authors who submitted their papers to this special issue and congratulate them for publishing their articles with *FOODS*. This would not be possible without the strong support of all devoted reviewers and their constructive comments, for which we are profoundly grateful. We would also like to acknowledge the 3D-SustJuice Project funded by the Croatian Science Foundation. Last but not least, we would like to thank Prof. Dr. Christopher John Smith, Editor-in-chief of *FOODS*, and entire MDPI team for their support of this special issue.
